# A proteolytic enzyme treatment to improve *Ulva laetevirens* and *Solieria chordalis* seaweed co-product digestibility, performance, and health in broilers

**DOI:** 10.1016/j.psj.2022.101777

**Published:** 2022-02-11

**Authors:** L. Stokvis, C. Rayner, M.M. van Krimpen, J. Kals, W.H. Hendriks, R.P. Kwakkel

**Affiliations:** ⁎ Wageningen University & Research, Wageningen Livestock Research, Wageningen 6708 WD, the Netherlands; † Wageningen University & Research, Animal Nutrition Group, Wageningen 6708 WD, the Netherlands

**Keywords:** broiler nutrition, digestibility, health, novel feed source, seaweed

## Abstract

To explore the potential use of seaweed co-products for broiler diets, this study investigates whether an enzyme treatment of seaweed co-products improves performance, in vivo digestibility and health in broilers. In total, 360 Ross 308 male broilers were fed one of 5 experimental diets: a basal diet, or a basal diet including the *U. laetevirens* or *S. chordalis* co-product, with or without proteolytic enzyme treatment of the seaweed, using 6 replicate pens of 12 birds each. The starter (d 0–13) and grower (d 14–21) diet contained 5 and 10% (w/w) seaweed product, respectively. A general linear model with contrast statements was used after model assumptions and goodness of fit were evaluated through normal distribution of residuals. Inclusion of seaweed in the broiler diets increased body weight gain (+14%; *P* = 0.002), and feed intake (+12%; *P* = 0.001) in the third week of the experiment. Birds fed the *U. laetevirens* compared to the *S. chordalis* diets had a higher body weight gain (+11%; *P* = 0.007), and a lower feed conversion ratio (FCR; -7%; *P* < 0.001). Seaweed inclusion reduced apparent pre-cecal digestibility of all nutrients (*P* < 0.05). Birds fed *U. laetevirens* vs. *S. chordalis* diets had a 10% reduced villus length (*P* < 0.001). Enzymatic treatment reduced the digestibility of most nutrients, and increased crypt depth in birds fed the *U. laetevirens* diets, whereas the opposite was observed for the birds fed the S*. chordalis* diets (Seaweed × Enzyme *P* = 0.035). Untreated vs. treated seaweed in the diets led to lower (−60%) plasma Interleukin-13 levels (*P* = 0.035). In conclusion, the proteolytic enzyme treatment of the seaweed co-products did not improve performance nor health-related parameters, and reduced digestibility of the diets. Dietary inclusion of *U. laetevirens* co-products did improve performance based on growth and FCR, whereas inclusion of *S. chordalis* did not. Inclusion of *U. laetevirens* in broiler diets slightly reduced duodenal villus length and crypt depth. The inflammation response was strongly reduced, specifically in birds fed the untreated *U. laetevirens* diet, making the *U. laetevirens* co-product of interest for future research.

## INTRODUCTION

To ensure a more sustainable broiler production, and to meet the increasing demand for animal derived foods, novel feed ingredients for broiler diets, especially protein sources, are receiving significant attention. Seaweed has several favorable characteristics as a protein source for animals, including a lack of required arable land or fresh water during production, as well as a current absence of significant competition as a food source. Additionally, seaweed can have a high protein content of up to 38% on DM basis and many species have a high concentration of important fatty acids (Biancarosa et al., 2017; Øverland et al., 2019) as for example the concentration of eicosapentaenoic acid (**EPA**) can be above 50% of the total fatty acid content (Santos et al*.*, 2017), although the fat content is generally below 40 g/kg DM in seaweed (Mišurcová, 2012).

Besides these positive characteristics, the current use of seaweeds as a feed ingredient also provides a number of challenges. For example, the nutritional composition of seaweed is highly variable, both within and between species, and content of minerals is relatively high with a range of 110 to 550 g/kg DM (Biancarosa et al., 2017; Øverland et al., 2019). The latter can be harmful when fresh seaweed is used as a feed ingredient, as for example excessive sodium content may lead to the overconsumption of water and consequently induce diarrhea (Koreleski et al., 2010). Total mineral content as well as content of specific minerals is highly variable between and within species. Moreover, untreated seaweeds are relatively poorly digested by monogastric animals with an apparent pre-cecal nitrogen digestibility of a maximum of 69% for *Saccharina latissima* products being reported in a recent broiler digestibility experiment (Bikker et al., 2020). Furthermore, fresh seaweed has a limited shelf life after harvest (Paull and Chen, 2008; Stévant et al., 2017). Currently inclusion of whole seaweed as a feed ingredient for simple stomached animals is not economically viable.

A biorefinery approach creating multiple fractions is suggested to overcome a number of the abovementioned drawbacks (Bikker et al., 2016; Torres et al., 2019; Bikker et al., 2020). For example, high value components or fractions can be extracted leaving a more cost-effective by/co-product for animal feed (Torres et al., 2019). Due to the low DM content of seaweed, pressing is used in the seaweed industry to fractionate resulting in a liquid fraction containing many soluble nutrients and cell contents, and the solid fraction. The latter is available for animal feed purposes, containing mostly cell walls and insoluble complexes. The currently available co-products are mainly available from the green seaweed *Ulva laetevirens* (previously *Ulva rigida*) and the red seaweed *Solieria chordalis*.

To be able to include any seaweed co-products in animal feed, the co-products need to have an acceptable mineral (i.e., salt) and heavy metal content (Besada et al., 2009; European regulations: EG 1334/2003 and European Commission, 2002), and a high nutrient digestibility for simple-stomached animals. The mineral content of seaweed or seaweed co-products can be reduced by washing with fresh water (Neveux et al., 2014), while improvement in nutrient digestibility might be achieved through the use of one or a combination of enzymes. Regarding the latter, multiple feed grade enzymes are currently commercially available such as proteolytic (Alcalase, Neutrase) and carbohydrytic enzymes (Ronozyme), each targeting specific molecular bonds. Although studies have been conducted using seaweed (co-products) in animal feed (e.g., Abudabos et al., 2013; Matshogo et al., 2020), little information is available on the effects of treatments aimed to increase the digestibility of seaweed co-products, or the effect of such treatments on the nutritional value of the seaweed co-products for broiler chickens (Krimpen and Hendriks, 2019). Simultaneously, there is a lack of knowledge of the effects of seaweed co-products on bird health, and whether these seaweed co-products are suitable, or even favorable, for inclusion in broiler diets. To assess the effect of feed ingredients on gastrointestinal health, intestinal pH and intestinal histological characteristics like villus height and crypt depth can be used to assess gastrointestinal functioning and uptake capacity. Furthermore, plasma cytokine levels can be used as an indicator of up- or downregulation of the immune response.

This study investigated whether a proteolytic enzyme treatment to seaweed co-products can improve performance and in vivo pre-cecal and total tract digestibility when included as protein source in a diet for broilers, and investigated effects on selected gastrointestinal functioning and health-related parameters (intestinal content pH, histology, plasma cytokine levels). The seaweed co-products tested were the remaining solid fractions after pressing and washing *U. laetevirens* and *S. chordalis*.

## MATERIALS AND METHODS

The animal experiment was conducted at the facility of Wageningen University & Research in Lelystad, the Netherlands. All experimental procedures were approved by the Animal Care and Use Committee of Wageningen University & Research, the Netherlands (AVD401002015196).

### Seaweed Harvesting and Processing

Both seaweed species (*U. laetevirens* and *S. chordalis*) were harvested from a beach in France near Guisseny on September 30, 2014 and Saint Hilaire de Riez on May 13 and 14, 2019, respectively, and harvested and processed by Olmix S.A. (Olmix Group, Bréhan, France). Upon harvest, *U. laetevirens* was immediately frozen and *S. chordalis* was directly processed. *Ulva laetevirens* was defrosted, ground to 50 to 1,000 nm particles (Inotec I175CDI-75D, Reutlingen, Germany) and pressed twice using a belt press (Flottweg BFRU 800, Vilsbiburg, Germany) at 6 bar with intermediate rehydration (DM = 196 g/kg) with fresh water, while *S. chordalis* was only pressed once (DM = 171 g/kg). The enzymatic treatment consisted of addition of 0.5% Alcalase (Soufflet Biotechnologies, Colombelles, France; ≥3,000 U/g) and Neutrase (Novozymes, Bagsværd, Denmark; 0.8 AU-N/g) to the seaweed cake (co-product) on a dry weight basis at 50°C for 5 h under low agitation, followed by a 5 min enzyme inactivation step at 90°C. Both untreated (−) and enzyme treated (+) seaweed co-products were air-dried at 60°C for 48 h followed by 24 h at 50°C up to 90% DM. Finally, all seaweed co-products were ground to pass a 1-mm sieve before inclusion in the experimental diets. The nutrient composition of the 4 seaweed co-products including their detailed mineral and amino acid (**AA**) composition is presented in Table 1.

**Table 1:** Analyzed nutrient content of untreated (−) and enzymatically^1^ treated (+) seaweed (*Ulva laetevirens* and *Solieria chordalis*) co-products.

Nutrient	*U. laetevirens*		*S. chordalis*	
Component	−	+	−	+
Gross nutrient content (g/kg dry matter)				
Dry matter (DM, g/kg)	943.6	942.3	942.3	919.5
Ash	151.8	151.3	328.7	321.0
Nitrogen (N)	49.1	49.4	38.2	35.7
Crude fat	16.5	19.6	5.6	5.8
Crude fiber	112.5	120.7	100.4	104.3
Sugar	4.1	5.0	16.7	13.7
Starch	19.5	21.1	20.1	8.3
Non starch polysaccharides^2^	562.7	556.1	437.7	472.6
Macrominerals (g/kg DM)				
Calcium	11.8	12.0	15.1	15.7
Phosphorus	2.0	2.0	2.4	2.5
Potassium	18.5	17.9	85.3	84.1
Sodium	8.8	8.6	13.1	12.9
Chloride	11.1	8.8	40.4	39.3
Magnesium	17.3	17.0	4.7	4.8
Sulfur	37.9	37.1	56.6	54.1
Microminerals (mg/kg DM)				
Iron	846	847	2165	2186
Copper	<5	5.4	8.6	10.2
Manganese	30	30	167	173
Zinc	32	29	74	72
Arsenic	12.0	11.2	7.7	7.7
Cadmium	0.29	0.31	0.26	0.29
Cobalt	0.4	0.4	1.2	1.3
Mercury	<0.01	<0.01	0.07	0.08
Lead	1.4	1.3	5.0	5.5
Nickel	4.8	4.7	5.2	5.9
Selenium	2.6	2.5	11.9	12.6
Amino acids (AA, g AA-N/100 g N)				
Lysine	6.1	6.0	4.8	5.0
Methionine	1.1	1.1	0.9	0.8
Cysteine	1.3	1.2	2.2	2.2
Threonine	3.6	3.5	2.8	2.6
Tryptophan	1.2	1.2	1.1	0.9
Leucine	4.5	4.3	3.6	3.2
Isoleucine	2.7	2.6	2.4	2.2
Histidine	3.5	3.6	3.9	3.7
Phenylalanine	2.7	2.6	2.2	2.1
Arginine	10.4	10.2	15.8	15.5
Asparagine + aspartic acid	10.5	10.3	8.2	8.0
Serine	4.0	3.9	2.7	2.5
Glutamine + glutamic acid	8.4	8.2	7.3	6.9
Glycine	6.8	6.7	6.7	6.5
Alanine	8.2	8.0	4.9	4.7
Valine	4.3	4.2	3.7	3.4
Proline	3.3	3.3	3.6	3.7
Tyrosine	1.5	1.5	1.7	1.6
Taurine	0.0	0.0	0.4	0.5
Total amino acids (g/kg DM)	276.6	273.2	193.6	174.2
Amino acid-nitrogen (g/kg DM)	41.2	40.7	30.1	27.2
Amino acid-nitrogen (g/100 g N)	83.9	82.5	78.8	76.2
Protein (g/kg DM)^3^	236.2	233.2	165.9	149.2
N:protein factor, K_p_^4^	4.81	4.72	4.34	4.18
N:protein factor, K_a_^5^	5.73	5.73	5.51	5.48

1 Alcalase (Soufflet Biotechnologies, ≥3,000 U/g) and Neutrase (Novozymes; 0.8 AU-N/g).

2 Calculated as NSP = 1,000–ash–(N-content × 5.0)–crude fat–(starch+sugars).

3 Sum of anhydrous amino acids.

4 Sum of anhydrous amino acids (g/kg DM) to nitrogen (g/kg DM) as per Mariotti et al. (2008).

5 Sum of anhydrous amino acids (g/kg DM) to amino acid-nitrogen (g/kg DM) as per Mariotti et al. (2008).

### Animals and Housing

A total of 360, one-day-old male broilers with an average BW of 44.4 ± 0.73 g (Ross 308, Probroed and Sloot, Groenlo, the Netherlands) were randomly assigned to one of 30 pens with 12 birds per pen. The average bird weight of each pen was kept within a 3% difference from the average pen weight. Each pen (1.5 m × 1.0 m) had a solid floor covered with wood shavings. All birds were vaccinated against infectious bronchitis at arrival, and against Newcastle disease at d 13. At d 16, the bedding material and solid floors were removed and replaced with slatted floors to enable collection of excreta. Each pen was assigned to one of 5 treatments in a completely randomized block design with 6 replicate pens per treatment. Ambient temperature was maintained at 32°C for the first 3 d and thereafter was gradually reduced to 22°C on d 21. A 23L:1D photoperiod was applied on the day of arrival, where after the dark period was increased by 1 h every day until a 16L:8D light schedule was achieved. Birds had ad libitum access to feed and water. At the end of the experiment at d 21, all birds were euthanized by an intravenous sodium pentobarbital injection in the wing vein.

### Experimental Diets

Starter (d 0–13) and grower (d 14–21) diets were formulated to meet or exceed the nutrient requirements for broilers (CVB, 2019), with the exception of protein and amino acids, in order to ensure treatment effects of these nutrients on growth performance. The grower diet was supplemented with 5 g/kg titanium (**Ti**) dioxide as an indigestible marker to allow determination of digestibility values. All diets were produced by Research Diet Services (Wijk bij Duurstede, the Netherlands) and fed as pellets (starter: 2.5 mm, grower: 3.2 mm). The seaweed containing diets consisted of the basal diet with either 5 (starter) or 10% (grower) seaweed co-product. The ingredients of the diets and analyzed nutrient composition of the grower diets are presented in Tables 2 and 3, respectively.

**Table 2:** Composition of the basal and untreated (−) and enzymatically^1^ treated (+) seaweed (*Ulva laetevirens* and *Solieria chordalis*) co-product containing starter (d 0 to 13) and grower (d 14 to 21) diets for broilers.

Ingredient (g/kg)	Starter diet					Grower diet				
	Basal	*U. laetevirens*		*S. chordalis*		Basal	*U. laetevirens*		*S. chordalis*	
		−	+	−	+		−	+	−	+
Maize starch	552.3	524.3	524.3	524.3	524.3	600.0	543.1	543.1	543.1	543.1
Soybean meal (CP > 480 g/kg)	200.0	189.8	189.8	189.8	189.8	120.0	108.7	108.7	108.7	108.7
*Ulva laetevirens*	-	50.0	50.0	-	-	-	100.0	100.0	-	-
*Solieria chordalis*	-	-	-	50.0	50.0	-	-	-	100.0	100.0
Oat hulls	80.0	75.9	75.9	75.9	75.9	80.0	72.4	72.4	72.4	72.4
Dextrose	50.0	47.4	47.4	47.4	47.4	55.4	50.2	50.2	50.2	50.2
Casein	36.3	34.4	34.4	34.4	34.4	61.5	55.7	55.7	55.7	55.7
Soya oil	19.4	18.4	18.4	18.4	18.4	23.8	21.6	21.6	21.6	21.6
Monocalcium phosphate	17.5	16.6	16.6	16.6	16.6	9.8	8.9	8.9	8.9	8.9
Chalk	8.4	8.0	8.0	8.0	8.0	12.6	11.4	11.4	11.4	11.4
Premix^2^	5.0	5.0	5.0	5.0	5.0	5.0	5.0	5.0	5.0	5.0
Titanium dioxide	5.0	5.0	5.0	5.0	5.0	5.0	5.0	5.0	5.0	5.0
Potassium carbonate	3.8	2.0	2.0	2.0	2.0	6.9	2.8	2.8	2.8	2.8
Sodium bicarbonate	2.5	3.0	3.0	3.0	3.0	2.3	0.4	0.4	0.4	0.4
Magnesium oxide	0.5	-	-	-	-	1.0	-	-	-	-
Salt	2.7	-	-	-	-	2.8	-	-	-	-
DL-Methionine	4.1	3.9	3.9	3.9	3.9	3.7	3.4	3.4	3.4	3.4
L-Arginine	3.3	3.1	3.1	3.1	3.1	4.3	3.9	3.9	3.9	3.9
L-Lysine HCl	3.0	2.8	2.8	2.8	2.8	2.5	2.3	2.3	2.3	2.3
L-Threonine	2.1	2.0	2.0	2.0	2.0	-	-	-	-	-
L-Valine	1.8	1.7	1.7	1.7	1.7	1.4	1.3	1.3	1.3	1.3
L-Leucine	1.7	1.6	1.6	1.6	1.6	1.4	1.3	1.3	1.3	1.3
L-Isoleucine	0.6	0.6	0.6	0.6	0.6	0.6	0.5	0.5	0.5	0.5
Diamol	-	4.5	4.5	4.5	4.5	-	2.1	2.1	2.1	2.1
AME (MJ/kg)	12.30	11.80	11.80	11.80	11.80	12.85	11.90	11.90	11.90	11.90

1 Alcalase (Soufflet Biotechnologies, ≥3,000 U/g) and Neutrase (Novozymes; 0.8 AU-N/g).

2 Provided per kg of diet: vitamin A, 10,000 IU; vitamin D_3_, 2,500 IU; vitamin E, 50 mg; vitamin K_3_, 1.5 mg; vitamin B_1_, 2.0 mg; vitamin B_2_, 7.5 mg; vitamin B_6_, 3.5 mg, vitamin B_12_, 20 µg; niacin, 35 mg; D-pantothenic acid, 12 mg; folic acid, 1.0 mg; biotin, 0.2 mg; Fe, 80 mg; Cu, 12 mg; Mn, 85 mg; Zn, 60 mg; I, 0.8 mg; Se, 0.15 mg.

**Table 3:** Analyzed nutrient content of the basal and untreated (−) and enzymatically^1^ treated (+) seaweed (*Ulva laetevirens* and *Solieria chordalis*) co-product containing grower diets as fed to the broilers.

Nutrient	Basal	*U. laetevirens*		*S. chordalis*	
Component		−	+	−	+
Gross nutrient content (g/kg dry matter [DM])					
Dry matter (g/kg)	888.9	888.9	890.2	889.7	889.7
Ash	54.3	59.6	59.1	73.8	73.3
Nitrogen	23.2	26.0	26.4	25.6	25.3
Crude fat	22.8	21.3	21.2	18.9	21.5
Crude fiber	35.8	43.9	42.6	42.4	42.4
Sugars	62.8	59.0	59.8	60.9	54.4
Starch	588.3	537.0	533.2	546.7	531.1
Non-starch polysaccharides^2^	155.7	193.4	194.7	171.8	193.0
Titanium	3.9	3.9	3.9	3.9	4.0
Amino acids (g AA-N/100 g nitrogen)					
Lysine	8.4	8.3	8.4	8.1	7.9
Methionine	2.5	2.4	2.3	2.2	2.2
Cysteine	0.7	0.8	0.8	0.9	0.9
Threonine	2.8	2.8	2.7	2.7	2.6
Tryptophan	1.1	1.1	1.1	1.0	0.9
Leucine	5.1	4.9	4.9	4.7	4.6
Isoleucine	3.2	3.1	3.1	3.0	2.9
Histidine	4.8	4.4	4.6	4.6	4.3
Phenylalanine	2.5	2.5	2.5	2.4	2.3
Arginine	15.6	14.5	14.7	15.3	15.2
Asparagine + aspartic acid	8.2	8.4	8.4	8.0	7.6
Serine	3.8	3.4	3.2	3.4	3.4
Glutamine + glutamic acid	15.8	14.3	14.3	14.2	14.0
Glycine	3.2	3.9	4.0	3.8	3.5
Alanine	3.5	4.3	4.3	3.6	3.4
Valine	4.6	4.6	4.6	4.4	4.3
Proline	5.7	5.2	5.2	5.2	5.1
Tyrosine	1.9	1.8	1.9	1.9	1.8
Taurine	0.0	0.0	0.0	0.1	0.1
Total amino acids (g/kg DM)	144.2	157.0	160.0	151.1	145.9
Amino acid-nitrogen (g AA-N/kg DM)	21.7	23.5	24.0	22.9	22.1
Amino acid-nitrogen (g/100 g N)	93.4	90.7	91.1	89.4	87.1
Protein (g/kg DM)^3^	124.6	135.4	137.7	130.4	126.0
Nitrogen:protein factor, K_p_^4^	5.37	5.22	5.22	5.10	4.97
Nitrogen:protein factor, K_a_^5^	5.75	5.75	5.73	5.71	5.71

1 Alcalase (Soufflet Biotechnologies, ≥3,000 U/g) and Neutrase (Novozymes; 0.8 AU-N/g).

2 Calculated as NSP = 1,000–ash–(N-content × 5.0)–crude fat–(starch+sugars).

3 Sum anhydrous amino acids.

4 Sum anhydrous amino acids (g/kg DM) to nitrogen (g/kg DM) as per Mariotti et al. (2008).

5 Sum anhydrous amino acids (g/kg DM) to amino acid-nitrogen (g/kg DM) as per Mariotti et al. (2008).

### Performance Measurements

Feed and water intake were monitored weekly per pen. Average BW per pen was determined upon arrival at the experimental facility, and again at d 7, 14, and 21. The feed conversion ratio (**FCR**) over a period was calculated as: total pen feed intake (**FI**) over the period/(pen BW end of period–pen BW start of period + pen BW of dead or culled birds) with FI per bird corrected for mortality calculated as: FCR × BW gain.

### Sample Collection and Chemical Analyses

Excreta were collected qualitatively from d 19 to 21, after which all birds were euthanized and ileal contents were collected from the distal 20 cm of the ileum, anterior to the ileocecal junction. Excreta and ileal digesta were stored at −20°C until further processing. Before chemical analyses, excreta and ileal digesta were freeze-dried, and all samples were ground using a 1 mm screen. All seaweed co-products and diets were analyzed for DM (ISO 6496, 1999), ash, (ISO 5984, 2002), nitrogen (N; ISO 5983, 2005), ether extract (crude fat; ISO 6492, 1999), fiber (ISO 6865, 2000), starch (ISO 15914, 2004), sugar (European Commission 152, 2009), tryptophan (ISO 13904, 2005), and other AAs (ISO 13903, 2005) as well as Ti (Short et al., 1996). Furthermore, Ca, P, Na, K, and Cl were analyzed (ISO 27085, 2009; ISO 6495, 2015). In the seaweed co-products, Fe, Mn, Mg, Zn, and Cu were analyzed (ISO 27085, 2009) as well as As, Cd, Pb, Hg, Co, Se, Ni, and S (DIN EN 15763, 2009). The ileal digesta samples were analyzed for DM, ash, N, AAs, and Ti, and the excreta samples were analyzed for DM, ash, fat, fiber, starch, sugar, and Ti. Organic matter (**OM**) was calculated as 1000–ash. Non-starch polysaccharide content (**NSP**) was calculated as 1,000–ash–(N-content × 5.0)–crude fat–(starch+sugars).

### Health-Related Parameters

At d 21, from 2 birds per pen with a BW close to the average pen BW, additional samples were collected. Before euthanasia, a blood sample was collected (4 mL) from the left wing vein for analysis of interleukin 13 (**IL-13**) and haptoglobin levels. Blood was centrifuged at 2,500 rpm for 15 min, the plasma (>500 µL) was transferred to Micronic tubes, and stored at -20°C pending analyses. ELISA kits specific for chicken haptoglobin (AbClonal, Woburn, MA) and IL-13 (Elabscience, Houston, TX) were used to determine cytokine levels according to the manufacturers’ instructions.

After euthanasia, full and empty gizzard weight was determined for potential effects of the seaweed co-products on gizzard development. The gizzard was separated from the proventriculus and the duodenum, and the full gizzard weighed. Gizzard contents were removed by rinsing with demineralized water and gentle drying using a paper towel before the empty gizzard was weighed.

Additionally, a duodenum sample (∼1 cm) was collected from the proximal duodenum just before the loop around the pancreas for histological analyses. This tissue sample was rinsed in a physiological salt solution (0.9% NaCl) and stored in a phosphate buffered 10% formalin fixative at 4°C until further analyses. During slide preparation, the tissue samples were dehydrated with increasing amounts of ethanol, cut in rings of ∼3-mm thickness and embedded in paraffin (Leica TP1020 tissue processor, Leica Microsystems B.V., Amsterdam, the Netherlands). Per sample, six 5-µm thin tissue sections were stained using Mayer's hematoxylin and eosin standard staining protocols. Pictures were taken using a light microscope (Lyca DM6b) using LASX software (Leica Microsystems B.V.) to measure villi length, crypt depth and tunica muscularis thickness. From each sample, a maximum of 30 intact villi, 30 crypts, 6 cross sections and 60 muscularis layer thickness were measured, of which the average was taken as value per sample. Villus length was defined as the distance from the tip of a villus to the villus-crypt junction. Crypt depth was defined as the distance from the villus-crypt junction to the circular muscle layer. The tunica muscularis thickness was defined as the distance between the start of the circular muscle layer to the serosa. The villi length to crypt depth ratio was calculated.

The jejunum was separated from the duodenum and ileum. Jejunal content was gently squeezed out and stored in a 5 mL Eppendorf tube. The jejunal content of both birds per pen was pooled to obtain sufficient material to measure pH (Mettler Toledo Seven2Go pH Meter, Mettler-Toledo AG, Analytical CH-8603, Schwerzenbach, Switzerland).

### Calculations and Statistical Analyses

Performance parameters were calculated using FI and BW measurements over time. Apparent pre-cecal digestibility and apparent total tract digestibility of nutrients in the experimental diets were calculated, using Ti as a marker according to the following equation:

$$\text{DC}\left(X\right)=\left(1-\frac{\left[\text{Ti}\right]\text{diet}\times \left[X\right]\text{sample}}{\left[\text{Ti}\right]\text{sample}\times \left[X\right]\text{diet}}\right)\times 100$$

where DC(X) is the apparent digestibility coefficient of nutrient X in % and [Ti]_diet_, [Ti]_sample_, [X]_diet_, and [X]_sample_ are the concentrations of Ti and nutrient X in the diet and digesta or excreta sample in g/kg, respectively (De Vries and Gerrits, 2018). The apparent pre-cecal digestibility and apparent total tract digestibility of nutrients in the seaweed co-products were calculated applying the difference method (Kong and Adeola, 2014) assuming additivity.

Data were analyzed using SAS statistical software (version 9.4, SAS Institute Inc., Cary, NC).

For all, except for histological data, a general linear model with contrast statements was used to 1) determine differences between birds fed the basal diet and those fed the seaweed containing diets (basal diet vs. [*U. laetevirens-, U. laetevirens*+, *S. chordalis*- and *S. chordalis*+]), 2) determine the effect of the different seaweed species (.(*U. laetevirens*- and *U. laetevirens*+ diets) vs. (*S. chordalis*- and *S. chordalis*+ diets), 3) determine the effects the enzymatic treatment (*U. laetevirens*- and *S. chordalis*- diets) vs. (*U. laetevirens*+ and *S. chordalis*+ diets). For histological data, a similar approach was taken as described above for all parameters except for muscularis thickness. Because of the nonlinear distribution of residuals, a generalized linear model was used for the latter.

Model assumptions and goodness of fit were evaluated through normal distribution of residuals, with data points being removed based on the studentized residuals >3 standard deviations from the sample mean. Data were square root or log transformed when necessary. One pen (untreated *S. chordalis*) was excluded of the digestibility analysis based on an outlier test with studentized residuals >3 standard deviations from the sample mean. Data are presented as means unless stated otherwise, with differences among means with a probability < 0.05 considered significant.

## RESULTS

### Nutritional Composition

The *U. laetevirens* untreated and enzymatically treated co-product contained lower amounts of ash (15 vs. 32%) and more N (4.9 vs. 3.7%), AAs (27 vs. 18%), and NSP (56 vs. 46%) compared to the two *S. chordalis* co-products (Table 1). The mineral composition differed between seaweed species and was not majorly impacted by the enzymatic treatment. The *S. chordalis* co-products contained higher levels of almost all macro- and microminerals (including heavy metals), whereas the *U. laetevirens* co-products contain higher concentrations of magnesium and the heavy metals cadmium and arsenic. All analyzed microminerals in the seaweed co-products were well within the limitations based on the European regulations for ingredients in animal diets, except for iron (EG 1334/2003; European Commission 32, 2002). High iron levels of over 2,100 mg/kg DM were observed in the treated and untreated *S. chordalis* co-products. The AA composition differed slightly between *U. laetevirens* and *S. chordalis* with the enzymatic treatment having no major effect on any of the AAs. Low levels of taurine were observed in *S. chordalis*, but not in *U. laetevirens*.

*Solieria chordalis* containing diets had 24 and 35% more ash compared to *U. laetevirens* and the basal diets, respectively (Table 3). All seaweed containing experimental grower diets had more fiber (18–22%), NSP (10–25%), and N (9–14%) and less starch (7–9%) compared to the basal diet.

### Performance

Performance data are summarized in Table 4. In the first week of the experiment, FCR was higher for birds fed the seaweed diets compared to birds fed the basal diet (1.02 vs. 0.95; *P* = 0.006). Furthermore, the FCR was higher for birds fed the *U. laetevirens* compared to the *S. chordalis* diets (0.99 vs. 1.05; *P* = 0.014). In the second week, the same basal vs seaweed diet effect was observed for FCR (*P* = 0.008) as in wk 1. Contrary to the first week, however, FCR was lower for birds fed *U. laetevirens* compared to the *S. chordalis* diets (1.18 vs. 1.23; *P* = 0.001). Furthermore, an increased water intake was observed of birds fed the seaweed diets compared to those fed the basal diet (880 vs. 763 mL; *P* = 0.003) and for birds fed the *S. chordalis* diets compared to those fed the *U. laetevirens* diets (943 vs. 817 mL; *P* < 0.001). In the third week, BW gain was higher for birds fed the seaweed compared to those fed the basal diet (355 vs. 310 g; *P* = 0.002), and higher for birds fed the *U. laetevirens* compared to the *S. chordalis* diets (374 vs. 336 g; *P* = 0.007). Simultaneously, FI in the same week was higher for birds fed the seaweed diets compared to birds fed the basal diet (662 vs. 589 g; *P* = 0.001), while no effect of seaweed species was observed. For birds fed *U. laetevirens* compared to *S. chordalis* diets, the FCR was lower (1.81 vs. 1.94; *P* < 0.001). Water intake of birds fed the *S. chordalis* diets was higher than that of birds fed the *U. laetevirens* diets (1,392 vs. 1,171 mL; *P* = 0.003).

**Table 4:** Effects of 5% (wk 1 and 2) and 10% (wk 3) inclusion of untreated (−) and enzymatically^1^ treated (+) seaweed (*Ulva laetevirens* and *Solieria chordalis*) co-products in a broiler diet (basal) on performance parameters.

Period	Basal diet (B)	Seaweed diets (S)				SEM	*P*-values^3^			
Parameter^2^		*U. laetevirens*		*S. chordalis*			B vs. S	S	Enzyme (E)	S × E
		−	+	−	+					
D 0–6										
Body weight d0 (g)	44.5	44.4	44.5	44.7	43.9	0.1	0.348	0.684	0.265	0.171
Body weight gain (g)	135	126	133	144	126	2.8	0.719	0.389	0.398	0.060
Feed intake (g)	128	129	141	141	125	3.0	0.393	0.746	0.777	0.052
Feed conversion ratio (g/g)	0.95	1.03	1.06	0.98	0.99	0.011	0.006	0.014	0.322	0.698
Water intake (mL)	424	448	566	499	406	26.9	0.407	0.408	0.851	0.119
Water:feed (mL/g)	3.33	3.46	3.18	3.54	3.23	0.19	0.616	0.809	0.130	0.820
D 7–13										
Body weight d7	180	171	178	189	170	2.8	0.185	0.399	0.367	0.051
Body weight gain	326	321	324	322	303	3.6	0.354	0.193	0.300	0.154
Feed intake	378	379	380	396	370	4.6	0.761	0.738	0.261	0.208
Feed conversion ratio	1.16	1.18	1.17	1.23	1.22	0.007	0.008	0.001	0.590	0.831
Water intake	763	820	814	987	899	19.6	0.003	<0.001	0.152	0.215
Water:feed	2.02	2.16	2.14	2.49	2.43	0.04	<0.001	<0.001	0.341	0.623
D 14–21										
Body weight d14	505	492	502	511	473	6.1	0.272	0.725	0.315	0.087
Body weight d 21	815	872	869	845	811	9.7	0.074	0.070	0.413	0.473
Body weight gain	310	380	367	334	338	6.6	0.002	0.007	0.712	0.528
Feed intake	589	672	675	646	654	9.3	0.001	0.251	0.778	0.911
Feed conversion ratio	1.90	1.77	1.84	1.94	1.94	0.016	0.241	<0.001	0.116	0.135
Water intake	1,193	1,161	1,180	1,396	1,387	32.0	0.213	0.003	0.946	0.837
Water:feed	2.03	1.73	1.75	2.16	2.11	0.04	0.130	<0.001	0.785	0.548
D 0–21										
Body weight gain	771	827	825	801	767	9.7	0.144	0.071	0.421	0.485
Feed intake	1,094	1,180	1,194	1,184	1,146	14.0	0.020	0.498	0.723	0.422
Feed conversion ratio	1.42	1.43	1.45	1.48	1.49	0.006	<0.001	<0.001	0.004	0.632
Water intake	2,380	2,430	2,559	2,882	2,691	57.4	0.047	0.023	0.800	0.193
Water:feed	2.18	2.06	2.14	2.44	2.34	0.04	0.254	<0.001	0.918	0.086

n.a., not applicable.

1 Alcalase (Soufflet Biotechnologies, ≥3,000 U/g) and Neutrase (Novozymes; 0.8 AU-N/g).

2 Each value is based on 6 replicate pens with 12 (Basal diet, *U. laetevirens*-, *U. laetevirens*+, *S. chordalis*+ diet) or 7 (*S. chordalis*- diet) birds each.

3 Statistical contrasts: Basal vs. seaweed: Basal diet vs. (*U. laetevirens*-, *U. laetevirens*+, *S. chordalis*- and *S. chordalis*+ diets), Seaweed: (*U. laetevirens*- and *U. laetevirens*+ diets) vs. (*S. chordalis*- and *S. chordalis*+ diets), Enzyme: (*U. laetevirens*- and *S. chordalis*- diets) vs. (*U. laetevirens*+ and *S. chordalis*+ diets).

Over the entire experimental period, seaweed diets compared to the basal diet resulted in a higher FI (1,176 vs. 1,094 g; *P* = 0.020), FCR (1.46 vs. 1.42; *P* < 0.001), and water intake (2,641 vs. 2,380 mL; *P* = 0.047) of birds. The FCR was higher when fed the *S. chordalis* compared to the *U. laetevirens* diets (1.49 vs. 1.44; *P* < 0.001), and also higher for birds fed the enzymatically treated compared to untreated seaweed co-products (1.47 vs. 1.46; *P* = 0.004). The water intake was higher for birds fed the *S. chordalis* compared to the *U. laetevirens* diets (2,787 vs. 2,495 mL; *P* = 0.023).

### Nutrient Digestibility

For all nutrients, the apparent pre-cecal digestibility of the basal diet was higher compared to that of the seaweed containing diets (*P* < 0.001; Table 5). Several seaweed × enzyme effects were observed which in the majority of cases showed a lower apparent pre-cecal digestibility coefficient for birds fed the enzymatically treated *U. laetevirens* diet compared to birds fed the untreated *U. laetevirens* and both *S. chordalis* diets (*P* < 0.05 for N, methionine, cysteine, threonine, phenylalanine, leucine, valine, glutamine+glutamic acid, serine, and tryptophan). The type of seaweed affected the digestibility for most nutrients (including AAs; *P* < 0.05), where birds fed the *U. laetevirens* diets showed lower apparent pre-cecal digestibility values compared to birds fed the *S. chordalis* diets (*P <* 0.05), except for histidine, glycine, tyrosine, and proline (*P* > 0.05). When an enzyme effect was observed, for ash, N and multiple amino acids, birds fed the enzymatically treated seaweed containing diets showed lower apparent pre-cecal digestibility values compared to birds fed the untreated seaweed diets (*P* < 0.05). No differences in apparent total tract digestibility were observed between basal and seaweed diets. The apparent total tract digestibility of crude fiber and crude fat were increased in birds fed the *S. chordalis* vs. *U. laetevirens* diets.

**Table 5:** Effects of 10% inclusion of untreated (-) and enzymatically^1^ treated (+) seaweed (*Ulva laetevirens* and *Solieria chordalis*) co-products in a broiler diet (basal) on apparent pre-cecal and total tract nutrient digestibility.

	Basal diet (B)	Seaweed diets (S)				SEM	*P*-value^3^			
Digestibility^2^		*U. laetevirens*		*S. chordalis*			B vs. S	S	Enzyme (E)	S × E
Nutrient		−	+	−	+					
Apparent pre-cecal (%)										
Dry matter	81.4	75.8	74.9	76.2	75.9	0.46	<0.001	0.047	0.065	0.335
Organic matter	82.9	78.1	77.2	78.5	78.4	0.40	<0.001	0.018	0.096	0.263
Ash	54.3	39.8	38.3	46.4	44.4	1.11	<0.001	<0.001	0.009	0.648
Nitrogen	84.0	74.4	71.4	74.8	74.6	0.85	<0.001	0.002	0.006	0.016
Lysine	89.7	83.2	81.6	84.0	83.5	0.56	<0.001	0.003	0.012	0.200
Methionine	94.6	92.2	90.2	91.9	91.8	0.28	<0.001	0.002	<0.001	<0.001
Cysteine	65.4	52.1	47.3	44.6	47.2	1.45	<0.001	<0.001	0.165	<0.001
Threonine	81.1	69.2	65.6	70.3	70.7	1.03	<0.001	<0.001	0.028	0.012
Isoleucine	87.2	78.6	75.5	79.3	78.4	0.78	<0.001	0.005	0.002	0.070
Arginine	88.1	81.5	80.2	78.1	77.8	0.74	<0.001	<0.001	0.113	0.331
Phenylalanine	88.8	76.8	73.5	77.2	76.9	1.04	<0.001	0.009	0.009	0.037
Histidine	85.5	73.2	72.9	74.5	73.8	0.97	<0.001	0.170	0.542	0.739
Leucine	89.0	81.0	77.8	81.7	81.0	0.82	<0.001	0.003	0.002	0.048
Valine	87.0	79.3	76.9	80.1	79.8	0.67	<0.001	0.002	0.012	0.044
Alanine	85.0	69.6	66.8	74.4	72.8	1.22	<0.001	<0.001	0.002	0.348
Asparagine + aspartic acid	84.9	70.7	67.2	73.5	72.1	1.19	<0.001	<0.001	0.003	0.178
Glutamine + glutamic acid	90.9	83.4	80.9	84.0	83.9	0.67	<0.001	0.002	0.016	0.033
Glycine	78.5	63.4	60.3	63.2	60.9	1.34	<0.001	0.968	0.005	0.666
Serine	83.2	69.6	64.6	72.2	74.3	1.21	<0.001	<0.001	0.015	<0.001
Tyrosine	89.9	81.0	79.5	80.6	79.9	0.78	<0.001	0.932	0.051	0.444
Proline	90.7	83.0	81.6	81.5	82.0	0.69	<0.001	0.210	0.299	0.055
Tryptophan	84.8	75.7	70.8	76.3	75.3	0.91	<0.001	<0.001	<0.001	0.002
Apparent total tract (%)										
Crude fiber	8.5	10.7	9.1	12.7	11.8	1.05	0.348	0.005	0.096	0.667
Crude fat	83.5	80.0	77.1	82.3	84.4	0.69	0.053	<0.001	0.648	0.050
Starch	98.9	98.6	98.6	99.0	98.6	0.06	0.296	0.417	0.218	0.181
Sugar	86.4	86.9	84.0	86.9	84.0	0.46	0.365	0.893	0.008	0.996

1 Alcalase (Soufflet Biotechnologies, ≥3,000 U/g) and Neutrase (Novozymes; 0.8 AU-N/g).

2 Each value is based on 6 pens with 12 birds each (Basal diet, *U. laetevirens*-, *U. laetevirens*+, *S. chordalis*+ diet) or 5 pens (n = 5 based on outlier test) with 7 birds each (*S. chordalis*- diet).

3 Statistical contrasts: Basal vs. seaweed: Basal diet vs (*U. laetevirens-, U. laetevirens*+, *S. chordalis-* and *S. chordalis*+ diets), Seaweed: (*U. laetevirens-* and *U. laetevirens*+ diets) vs. (*S. chordalis-* and *S. chordalis*+ diets), Enzyme: (*U. laetevirens-* and *S. chordalis-* diets) vs. (*U. laetevirens*+ and *S. chordalis*+ diets).

The seaweed apparent digestibility data calculated by the difference method showed large variations (Table 6), mainly in the ash fraction and some of the AAs (e.g., cysteine, threonine and phenylalanine). For almost all nutrients, the apparent pre-cecal digestibility of *U. laetevirens* was higher (*P* < 0.05) than that of *S. chordalis* co-products. In addition, the enzyme treatment reduced the apparent pre-cecal digestibility of most nutrients (*P* < 0.05). The apparent total tract digestibility of crude fiber in *S. chordalis* was higher than that of *U. laetevirens*.

**Table 6:** Effects of 10% inclusion of untreated (−) and enzymatically^1^ treated (+) seaweed (*Ulva laetevirens* and *Solieria chordalis*) co-products in a broiler diet (basal) on seaweed co-product apparent pre-cecal and total tract nutrient digestibility.

Digestibility^2^	*U. laetevirens*		*S. chordalis*		SEM	*P*-value^3^		
Nutrient	−	+	−	+		Seaweed (S)	Enzyme (E)	S × E
Apparent pre-cecal (%)								
Dry matter	30.2	21.1	33.6	29.0	1.73	0.096	0.036	0.484
Ash	−16.3	−31.3	139.8	116.5	16.43	<0.001	0.005	0.493
Organic matter	31.0	22.1	20.1	17.7	1.80	0.022	0.075	0.312
Nitrogen	63.2	34.2	38.2	29.0	3.76	0.008	0.002	0.077
Lysine	65.4	47.8	27.3	21.1	4.20	<0.001	0.006	0.169
Methionine	66.0	43.0	31.9	21.1	3.69	<0.001	<0.001	0.003
Cysteine	63.5	12.6	33.5	49.9	5.77	0.615	0.043	0.001
Threonine	70.9	33.4	18.9	7.9	6.11	<0.001	0.002	0.077
Isoleucine	56.0	23.3	23.1	2.2	4.91	<0.001	<0.001	0.300
Arginine	52.8	37.8	37.9	24.6	3.24	0.010	0.012	0.863
Phenylalanine	61.1	26.3	9.4	−6.9	6.23	<0.001	0.001	0.169
Histidine	2.9	1.8	1.0	−18.2	4.02	0.140	0.220	0.255
Leucine	64.2	28.7	20.5	−2.2	5.84	<0.001	<0.001	0.279
Valine	81.7	55.4	41.6	27.4	4.92	<0.001	<0.001	0.241
Alanine	147.4	116.0	68.7	38.1	9.59	<0.001	<0.001	0.949
Asparagine + aspartic acid	53.4	17.5	17.1	−6.7	5.78	<0.001	<0.001	0.417
Glutamine + glutamic acid	24.6	−1.0	1.0	−7.2	3.53	0.007	0.003	0.105
Glycine	110.3	77.3	63.7	25.0	7.73	<0.001	<0.001	0.737
Serine	29.8	−21.9	−13.8	−4.1	5.14	0.059	0.002	<0.001
Tyrosine	47.6	31.1	28.3	13.7	3.62	0.002	0.009	0.857
Proline	31.7	19.1	2.9	3.4	3.36	<0.001	0.194	0.182
Tryptophan	85.8	38.3	51.3	12.7	6.27	<0.001	<0.001	0.423
Apparent total tract (%)								
Crude fiber	43.9	29.6	62.0	53.4	4.17	0.010	0.120	0.695
Crude fat	27.4	8.7	10.3	31.5	5.94	0.754	0.980	0.111
Starch	1.3	1.6	4.9	−1.4	0.84	0.991	0.078	0.039
Sugar	11.0	−16.4	29.6	−3.4	5.70	0.155	0.006	0.776

1 Alcalase (Soufflet Biotechnologies, ≥3,000 U/g) and Neutrase (Novozymes; 0.8 AU-N/g).

2 Each value is based on 6 pens with 12 birds each (Basal diet, *U. laetevirens*-, *U. laetevirens*+, *S. chordalis*+ diet) or 5 pens (n = 5 based on outlier test) with 7 birds each (*S. chordalis*- diet).

3 Statistical contrasts: Seaweed: (*U. laetevirens-* and *U. laetevirens*+ diets) vs. (*S. chordalis-* and *S. chordalis*+ diets), Enzyme: (*U. laetevirens-* and *S. chordalis-* diets) vs. (*U. laetevirens*+ and *S. chordalis*+ diets).

### Health-Related Parameters

No significant differences were observed in empty or full gizzard weight and gizzard content between treatments (Table 7). Numerically, empty gizzard weight, full gizzard weight, and gizzard content were 7, 12, and 24 % lower in birds fed the enzymatically treated seaweed diets compared to birds fed the untreated seaweed containing diets, respectively. Compared to birds fed the *S. chordalis* diets, birds fed the *U. laetevirens* diets had an 11% lower (*P* < 0.001) villus length and a 10% lower (*P* = 0.006) villus length to crypt depth ratio (Table 7). Birds fed the treated *U. laetevirens* diet had an 8% larger crypt depth compared to birds fed the untreated *U. laetevirens* diet, whereas the opposite was observed for the treated and untreated S*. chordalis* diets (−4%; Seaweed × Enzyme effect, *P* = 0.035). No significant differences were observed in jejunal digesta pH (Table 7) or plasma IL-13 and haptoglobin levels (Table 7) between the basal and seaweed diets or between the seaweed diets. An enzyme effect (*P* = 0.035) was observed for plasma IL-13 concentration with the enzyme treatment leading to higher values.

**Table 7:** Effects of 10% inclusion of untreated (−) or enzymatically^1^ treated (+) seaweed (*Ulva laetevirens* and *Solieria chordalis*) co-products in broiler diet (basal) on gastrointestinal tract (GIT) characteristics and plasma cytokine levels.

Tissue	Basal diet (B)	Seaweed diets (S)				SEM	*P*-value^4^			
Parameter		*U. laetevirens*		*S. chordalis*			B vs. S	S	Enzyme (E)	S × E
		−	+	−	+					
Gizzard^2^ (g/kg BW)										
Weight empty	15.6	15.4	14.4	15.5	14.4	0.13	0.332	0.897	0.135	0.966
Weight full	22.2	21.7	18.6	22.5	21.0	0.13	0.394	0.293	0.128	0.615
Content	6.6	6.3	4.2	7.0	6.5	0.13	0.531	0.130	0.190	0.425
Duodenum^2^										
Villus length *(µm)*	1,818	1,628	1,624	1,782	1,820	24.2	0.071	<0.001	0.715	0.659
Crypt depth *(µm)*	84	76	82	80	77	1.1	0.033	0.871	0.592	0.035
Villus length:crypt depth	22	22	20	22	24	0.4	0.792	0.006	0.948	0.063
Muscularis thickness *(µm)*	132	124	129	130	117	3.0	0.452	0.634	0.600	0.181
Jejunum^3^										
Digesta pH	6.20	6.10	6.10	6.10	6.10	0.03	0.097	0.782	0.963	0.872
Blood plasma^2^										
Interleukine-13 (pg/mL)	22.4	9.1	19.6	18.0	23.8	2.57	0.921	0.267	0.035	0.723
Haptoglobin (ng/mL)	2.0	2.1	1.3	1.7	1.5	0.15	0.438	0.928	0.097	0.521

1 Alcalase (Soufflet Biotechnologies, ≥3,000 U/g) and Neutrase (Novozymes; 0.8 AU-N/g).

2 Each value is based on 6 replicate pens with 2 birds per pen.

3 Each value is based on 6 replicate pens with 1 pooled sample of 2 birds per pen.

4 Statistical contrasts: Basal vs. seaweed: Basal diet vs. (*U. laetevirens*-, *U. laetevirens*+, *S. chordalis* and *S. chordalis*+ diets), Seaweed: (*U. laetevirens-* and *U. laetevirens*+ diets) vs. (*S. chordalis-* and *S. chordalis*+ diets), Enzyme: (*U. laetevirens-* and *S. chordalis*- diets) vs. (*U. laetevirens*+ and *S. chordalis*+ diets).

## DISCUSSION

### Nutritional Composition

As expected, the enzymatic treatment of the seaweed co-products did not cause large differences in nutritional composition, whereas large differences between the 2 seaweed species were observed in ash, N, fat, fiber, and sugar content. The high mineral content of *S. chordalis* co-products and the concomitant effect on water intake might have affected other observed parameters. For example, the higher water intake could lead to diarrhea, suboptimal bird health and reduced performance (Guiry and Blunden, 1991; Koreleski et al., 2010). Signs of diarrhea were not observed in our study, although excreta water content was influenced and will be addressed later in this discussion. The iron content exceeded the maximum dietary level of European regulations for animal diets, while no separate regulations are in place for maximum iron content in dietary ingredients. With an inclusion level of 5 and 10%, the iron content of the seaweed co-products is diluted to dietary levels within the European regulation limits. Mineral content in seaweed depends on amongst others seaweed species and environmental factors (Boderskov et al., 2016; Sharma et al., 2018). Due to this large variation the mineral level of seaweed to be included in animal diets needs to be determined accurately. Furthermore, the ratio between (trace) minerals is important to take into account (Bao and Choct, 2009). For example, iron impairs zinc absorption (Solomons and Jacob, 1981), potentially leading to a zinc deficiency and consequently a depressed growth performance and animal welfare, while it met dietary specification for practical diets (CVB, 2019). The N-content varied considerably between the co-products of the 2 species, although due to the low inclusion level of 5 and 10%, dietary N (and AA) intake per kg DM only differed 1.5 to 3%.

Based on their gross nutritional composition, *U. laetevirens* co-products are considered more valuable feed ingredients for broiler nutrition compared to *S. chordalis* co-products due to the higher AA and true protein, and lower ash content.

### Performance

The higher FI of birds fed the seaweed containing diets compared to birds fed the basal diet, and of birds fed *U. laetevirens* vs. *S. chordalis* diets might be explained by differences in calculated ME content and nutrient digestibilities between the diets, as could be a result of higher levels of dietary fiber. This may have led to a compensatory FI which consequently led to an increased protein intake of 14.7 to 15.9 vs. 12.1 g N in the third experimental week of birds fed the seaweed vs. basal diets Gous et al. (2018). found an inverse relationship between protein content and FI of a diet, depending on the ME to digestible protein ratio. Taking into account the N digestibility, digestible N intake was 11.0 to 11.6 vs. 10.2 g N per bird of broilers fed the seaweed vs. basal diets in the third experimental week, respectively, indicating the ME and digestible protein intake were similar between treatment groups.

Based on a higher FCR in the first week and a lower FCR in the third week of birds fed *U. laetevirens* co-products compared to the other dietary treatments, it appears that these birds had to adjust to these seaweed co-products. Remarkably, the best FCR in the third week was observed for the untreated *U. laetevirens*, although this is not reflected in the digestibility coefficients. Contrary to the birds fed the *S. chordalis* diets, water intake of birds fed the *U. laetevirens* diets was not increased with FI.

The relatively large water intake of birds fed the seaweed diets might have been caused by the dietary electrolyte balance. Water intake was indeed correlated with ash content of the diets, with especially the birds' fed *S. chordalis* diets having a higher water intake Koreleski et al. (2010). also observed changes in water intake and DM content of excreta as a response to changing dietary levels of specific minerals. In the current study, the excreta moisture content of birds fed the basal diet was 743 g/kg, similar to 768 and 724 g/kg of birds fed the *S. chordalis* diets. Lower moisture levels of 683 and 662 g/kg were observed in excreta of birds fed the *U. laetevirens* diets. This is contrary to their water intake, which was similar for birds fed the *U. laetevirens* diets compared to the basal diet in the third experimental week. It must be mentioned that the collection method of excreta was not designed for precise excreta moisture determination, and these data reflect differences between treatments rather than precise absolute values. Differences in water intake and excreta moisture content in the current study might also be related to changes in digesta viscosity caused by differences in soluble NSP (Francesch and Brufau, 2004). Viscoelastic properties of digesta were, however, not analyzed in this experiment.

Literature on the effects of seaweed (co-product) inclusion in broiler diets at nutritionally significant levels (>5%) on broiler performance is scarce and results are inconsistent. In one study, 1 to 3% green seaweed *Ulva lactuca* was added to broiler diets from d 12 to d 33 at the expense of corn (Abudabos et al., 2013). Performance parameters were not influenced by the seaweed inclusion, although another study reported severe negative effects on performance after inclusion of 10, 20, and 30% *Ulva rigida* seaweed in broiler diets and conclude that this intact seaweed is not suitable as dietary ingredient at levels of 10% or higher (Ventura et al., 1994). These differences can at least partly be attributed to the different inclusion levels and the use of different seaweed species, since large differences in chemical composition exist between and within seaweed species (Biancarosa et al., 2017; Sharma et al., 2018).

### Digestibility

The enzymatic treatment reduced nutrient digestibility in the diets and bird performance. To our knowledge, very few studies have evaluated the nutrient digestibility of seaweed, or seaweed products in poultry, or the effect of an enzyme treatment on nutrient digestibility of those seaweed products. In seabass, apparent nutrient digestibility of complete (not fractionated) *U. rigidi* seaweed and diets was increased after treatment with an enzymatic cocktail consisting of lipase, pectinase, cellulase, and amylase, although still lower than that of a diet without seaweed. Even though, it was hypothesized that this was likely due to increasing accessibility to proteins for intrinsic proteases due to disrupting other seaweed cell structures (Batista et al*.*, 2020). Furthermore, these authors observed an interaction between the seaweed and diet digestibility, and discuss the release of complex polysaccharides that impaired nutrient digestibility. *Ulva* spp. inclusion up to 3.5% (w/w) in isonitrogenous and isocaloric diets of Boschveld indigenous hens did not alter nutrient digestibility of seaweed containing diets (Nhlane et al*.*, 2020). In the present study, nutrient digestibility may have been reduced by for example complex forming and precipitation of free AAs with heavy metals (Ashmead, 1992). Furthermore, the enzyme treatment might have altered the dietary and consequently intestinal content viscosity, as also hypothesized by Batista et al*.* (2020), which is known to by itself lead to reduced nutrient digestibility and impaired growth performance (Smits et al., 1997). The enzyme treatment might additionally have led to more hydrophobic interactions in the enzyme treated seaweed co-products, which are known to reduce protein digestibility in proso millet flour and rice (Gulati et al., 2017; Liu et al., 2019). Based on this experiment, we cannot conclude what mechanism(s) has/have caused the reduced digestibility as a result of the enzyme treatment.

Digestibility values of the individual seaweed co-products were calculated using the difference method, which assumes an absence of interactions between the feed ingredient of interest and the basal diet (Kong and Adeola, 2014). Regarding the high variation in the digestibility values of these seaweed co-products, this assumption may not be valid as digesta viscosity and microbiota composition may be different due to the diets. When using the difference method, a high inclusion level of the seaweed co-product in the basal diet is desirable as this will lead to a more precise determination of actual digestibility values of the ingredient. Lower ingredient inclusion levels increase the error of the digestibility estimate and a potential greater deviation from the actual digestibility value if the ingredient is included in diets as the sole protein source. It, however, remains important to evaluate the effect of the seaweed (co-)products on nutrient digestibility when included in a practical poultry diet.

### Health-Related Parameters

The lack of differences observed in gizzard characteristics and jejunal pH was unexpected, since gizzard weight and the pH in most parts of the digestive tract change with a change in fiber source (Jiménez-Moreno et al., 2009). Differences in gizzard development are most often ascribed to diet structure instead of composition (Svihus, 2011; Hamungalu et al., 2020), although these factors did not differ among treatment groups in such a way that it affected gizzard characteristics in the present study. Haptoglobin is associated with iron binding and oxygen transport by red blood cells. The high iron levels in the seaweed diets, especially in the *S. chordalis* diets, did not translate into differences in plasma haptoglobin levels.

Increased villi length corresponds to a higher nutrient uptake capacity (Cañedo-Castro et al., 2019), whereas deeper crypts are indicative of a higher villi cell turnover rate, associated with a reduced digestion and uptake capacity (Pluske et al*.*, 1996). Consequently, a higher villus length to crypt depth ratio indicates a slower turnover rate of intestinal cells leading to lower maintenance requirements and potentially increased efficiency of animals. Villi length tended to decrease by including seaweed in broiler diets. Combined with the decreased crypt depths of birds fed the seaweed diets vs the basal diet, this indicates that less energy is spent on maintenance of the intestinal lining by the birds fed seaweed diets. However, this did not result in a better performance. In contrast, when broilers were fed 2, 4, or 6% *U. rigida* (washed with fresh water, sun-dried and ground), longer villi were observed compared to a diet without seaweed, with the highest length observed in the chickens fed the 2% diet (Cañedo-Castro et al., 2019). These authors combined their finding with lower serum cholesterol and triglyceride levels, without negative effects on performance. They suggested this was potentially caused by either sulphated polysaccharides or fatty acids from the seaweed product and conclude that *U. rigida* would be a good pre-biotic for enhancement of broiler health.

Birds fed the *U. laetevirens* vs. *S. chordalis* diets had shorter villi and a decreased villus height to crypt depth ratio, which does not reflect the better performance results of birds' fed *U. laetevirens* diets. In the literature, an increase in villi length between treatments is explained by a need for an increased absorption area in order to digest and absorb nutrients from more viscous intestinal contents in a diet with higher NSP levels (Van Krimpen et al., 2015). Indeed, in our study the NSP content of the *S. chordalis* co-products was higher compared to that of the *U. laetevirens* co-products, and the villi length was increased in the former compared to the latter.

The higher IL-13 levels in the birds fed enzymatically treated vs untreated seaweed diets indicate a stronger anti-inflammatory response to extracellular pathogens. This effect was twice as large in birds fed *U. laetevirens* diets (54% reduction) compared to birds fed *S. chordalis* diets (24% reduction). Common dietary ingredients like soy contain a relatively large amount of NSP which are mostly indigestible for poultry. Part of this NSP fraction like mannans and galactomannans, have membrane components similar to that of pathogens, triggering a feed induced immune response (a.o Kogut, 2017.). Hence, the observed IL-13 level in the control and enzymatically treated diets might indicate an increased inflammatory response. Moreover, the lower IL-13 levels in the birds fed untreated seaweed diets might indicate that these untreated products improve gut health, but that the enzymatic treatment diminishes this positive effect. Potentially, proteins or peptide-carbohydrate complexes cause this positive effect, while the proteolytic enzymes reduce these bioactive complexes to ineffective building blocks.

### Recommendations

To unravel the working mechanism of seaweed products and the effects on broiler health, further studies need to be conducted. A suggested focus is toward the effects of those products on viscous characteristics of the diets and chyme. Furthermore, the effects of the polysaccharides and NSP on broilers, for example by analyses of microbiota in the ceca, are of interest. An enzyme treatment with a carbohydrase targeting specific seaweed polysaccharides is suggested to improve digestibility and nutritional value of seaweed for broilers, although this enzyme should be tailored to the seaweed species of focus as different carbohydrates are present among seaweed species.

## CONCLUSIONS

This study confirms the high mineral content of *U. laetevirens* and *S. chordalis* co-products and their relatively poor nutrient (especially protein and amino acid) digestibility in broilers. The inclusion of *U. laetevirens* and *S. chordalis* co-products in experimental broiler diets reduced the overall nutrient digestibility of the diet, with the proteolytic enzyme treatment of seaweed co-products reducing rather than improving performance. Addition of the *U. laetevirens* co-products to a basal diet improved performance based on growth and FCR. The enzyme treatment did not improve the studied health-related parameters, whereas the untreated seaweed products might improve broiler health.
